# Editor's choice: AHS reaches leaps and bounds

**DOI:** 10.4314/ahs.v25i1.1

**Published:** 2025-03

**Authors:** James K Tumwine

In this March 2025 issue of *African Health Sciences*, we have a selection of papers on infectious diseases, non-communicable diseases (NCDs.) and others. Hence, Iqbal^1^, as well as Sudanese researchers^2^, give us a glimpse into antimicrobial resistance and so does Mert^3^, who describes surgical site infections. The infectious disease theme is also echoed by a case report of an unusual abscess^4^. The role of *Toxoplasma gondii* in degenerative conditions is discussed^5^. Algerian scientists, who also studied *Toxoplasma gondii*, highlight its prevalence and risk factors in pregnant women^6^. The next paper helps us to understand *Blastocytosis spp* regarding clinical presentation and irritable bowel^7^. COVID-19 is not back, but we continue to reflect on it. For example, researchers from Nigeria have written for us an interesting paper on stress and coping with the pandemic^8^. On the other hand, a report from northern Uganda highlights the optimization of service delivery for contraceptives among adolescents living with HIV^9^.

Continuing the infectious disease theme, Ihekaike and others^10^, describe kidney diseases among children living with HIV, while Mirambo and colleagues report on mumps among the un vaccinated in Northern Tanzania^11^. Hepatitis B and C continue plaguing our continent. Thus, Nigerian researchers report on the factors associated with hepatitis B and among children with sickle cell anemia^12^. Pneumonia on the other hand remains an important cause of morbidity and mortality, and in this vein, Ye and colleagues report on pneumonia in children^13^. Adekemi and others^14^, provide us with a protocol for systematic reviews on diagnostic references in Africa. The section ends with several papers on arthritis.^15^-^16^.

This pushes us into the realm of the ever-ubiquitous non-communicable diseases (NCDs) which have reached endemic levels. Hence, we have papers on myocardial ischemia^17^, acute coronary syndrome^18^, and aortic stenosis^19^. Others are on subarachnoid bleeding^20^; cardiovascular diseases and contraceptives^21^. Work on the rehabilitation of stroke patients^22^ ends this cardiovascular disease section. Next, we have papers on breast cancer^23^, metastases in the peritoneum^24^, management of post cystectomy strictures^25^, and gastrointestinal malignancies in Nigeria^26^.

The NCD theme continues with papers on renal diseases: care of post renal transplant patients^27^, distribution of kidney disease in Africa^28^, acute kidney injury in Kenya^29^, and diabetic nephropathy^30^. There are papers on intake of fruits and vegetables^31^, effect of weight reduction in patients with asthma and obesity^32^; and a unique study on dentures^33^. A paper on psoriasis^34^; the immunology of sickle crises^35^; and frailty of the eldery ends this section on non-communicable diseases.^36^ The next section is on reproductive health and sexuality issues.

This is ushered in by papers on adolescent attitude to premarital sex^37^; low antenatal care visits^38^; perimenopause^39^; post menopause^40^; contraception^41^, and cesarean section scars^42^.

We have a few papers on anesthesia for hernia repair^43^; neuro tube defects^44^, and adenoidectomy^45^. We have papers on temporomandibular joint in Brazil^46^, surgical outcomes in LMICs^47^. The final paper is on vertebroplasty for fractures of the spine.^48^

We wish you enjoyable reading of this bumpy issue of *African Health Sciences*.

## References

[1] Iqbal R, Ahmad P, Tahir Z (2025). Antimicrobial susceptibility pattern of pathogens isolated from surgical wound infections in tertiary care hospitals of Pakistan. Afri Health Sci.

[2] Hamadalneel YB, Ahmed HO, Alamin MF (2025). Patterns of bacterial pathogens and their antimicrobial susceptibility from blood culture specimens in Wad Medani, Sudan: a four-year laboratory-based, cross-sectional study. Afri Health Sci.

[3] Adali M (2025). Surgical site infections following emergency abdominal surgery: a prospective cohort study. Afri Health Sci.

[4] Suo X, Chen J, Wang S, Zhang K (2025). Abscess of the ligamentum teres hepatis and Arantius' ligament: a case report. Afri Health Sci.

[5] Samadzade R, Maçin S, Nsangou AM, Gumus H, Akdam N, Guney F (2025). Possible link between *Toxoplasma gondii* and neurodegenerative diseases. Afri Health Sci.

[6] Dahmani A, Aiza A, Zenia S (2025). Seroprevalence and associated risk factors of *Toxoplasma gondii* among pregnant women in Ain Defla, northwest Algeria. Afri Health Sci.

[7] Feride Ş, Rugıyya S, Hüseyin K, Salih M (2025). Investigation of *Blastocystis spp*. in patients with inflammatory bowel disease by direct microscopy and molecular methods. Afri Health Sci.

[8] Agbonselohbor B, Aslanoğlu A, Bilgiç N, Alsharawneh A, Elshatarat RA, Saleh ZT (2025). Associated factors of Nigerian Nurses' Emotion regulation, perceived stress, and coping mechanism during COVID-19 Pandamic: a cross-sectional study. Afri Health Sci.

[9] Ajilong H, Bongomin F, Pebolo PF, Obol JH (2025). Differentiated service delivery strategies to optimize modern contraceptive uptake among adolescents with HIV in Northern Uganda. Afri Health Sci.

[10] Ihekaike MM, Shehu M, Mbah IO, Kanhu PU, Shehu H, Isichei CO (2025). Kidney Disease in HIV Infected Children on HAART in Nigeria. Afri Health Sci.

[11] Mirambo MM, Nyawale H, Kalatwa AB, Msemwa B, Mshana SE (2025). High seropositivity of Mumps virus IgG antibodies in unvaccinated population of Mwanza, Tanzania: A community-based study. Afri Health Sci.

[12] Nwukor SA, Ezeonu CT, Orji M-LC, Udechukwu PN, Omeje NK (2025). Prevalence and correlates of Hepatitis B and C infections in Sickle cell anaemic (SCA) children compared to Controls in a Tertiary Hospital, Abakaliki, Southeast, Nigeria. Afri Health Sci.

[13] Ye W, Wu J, Cao M, Yang Z (2025). Establishment and validation of a predictive model for severe pneumonia in children. Afri Health Sci.

[14] Adekanmi AJ, Akinlade BI, Adedoyin OA (2025). Diagnostic reference levels in Africa: A protocol for systematic review and meta-analysis. Afri Health Sci.

[15] Yi X, Lei J, Hua Y, Chen C, Yang J (2025). MicroRNA-9-5p regulates apoptosis of human osteoarthritis chondrocytes through suppressing TnC. Afri Health Sci.

[16] Guo J, Ma X, Wu L, Zhao W, Zhang Y, Hao C (2025). Inhibition of Nuclear factor kappa B ameliorates bone destruction and osteoclastogenesis in collagen-induced arthritis mice. Afri Health Sci.

[17] Lu Y, Wang F, Xiao N, Fan Z, Xiong L, Kou J (2025). Mild hypothermia (35°C) reduces myocardial ischemia-reperfusion injury and attenuates hypoxia induced apoptosis of H9C2 cardiomyocytes by changing the phosphorylation level of Connexin43 (Cx43) protein. Afri Health Sci.

[18] Buyukkilic BZ, Eroglu SA (2025). Assessment of the complaints and the hospital application of patients with acute coronary syndrome, and the patient's knowledge and behaviors regarding the management of cardiovascular risk factors. Afri Health Sci.

[19] An J, Shi F, Wang H, Zhang H, Liu S (2025). The efficacy and safety of transcatheter aortic valve replacement in elderly patients with aortic stenosis. Afri Health Sci.

[20] Wang X, Lin Z, Jin D, Huang R, Kang T, Su W (2025). Value of cerebrovascular hemodynamics and thromboelastogram-related parameters in evaluating the effect of Naomai Jiejing Decoction on the rehabilitation of patients with aneurysmal subarachnoid hemorrhage. Afri Health Sci.

[21] Iya B, Akpan U, Nja G, Enang E, Adoga O, Etokidem A (2025). Anti-oxidative enzymes, cardiovascular disease risk factors, and adipokines in Nigerian women on oral and implant contraceptives. Afri Health Sci.

[22] Duo Y, Zhong G, Shen Z (2025). Effect of IIFAR information nursing model and comprehensive rehabilitation training on negative emotions and rehabilitation outcomes in elderly hemiplegic stroke patients. Afri Health Sci.

[23] Zhou X, Yu M, Xie X (2025). Effects of ultrasound-guided nerve block combined with PCIA analgesia on postoperative pain, inflammatory response, hospital stay, and adverse reactions in breast cancer surgery. Afri Health Sci.

[24] Gu X, Li Y, Shi G, Yang L, Yang Y, Zhang Z (2025). Diagnostic Value of the Cardiophrenic Lymph Nodes in Gastric Peritoneum Metastases. Afri Health Sci.

[25] Liu N, Xing L, Chen H, Chen S, Li M, Zhang X (2025). Sequential dilatation of two balloons and double D-J stents for therapy of ureteroenteral anastomotic stricture in patients following radical cystectomy and Bricker urinary diversion. Afri Health Sci.

[26] Tagar E, Irabor D, Kpolugbo J, Owobu C, Chukwu I (2025). Pattern of gastrointestinal malignancies in a suburban centre in Southern Nigeria. African Health Sciences.

[27] Wang Y, Ding P, Yang Q, Xia M, Li N, Zhou L (2025). Effect of nursing procedure-oriented comprehensive nursing on negative emotion and self-care ability of renal transplant recipients after operation. Afri Health Sci.

[28] Nweke M, Ado-Aghughu T, Daniels T, Imo U (2025). Burden and distribution of chronic kidney disease in sub-Saharan Africa: a systematic review with meta-analysis. Afri Health Sci.

[29] Demba RN, Aradi SM (2025). Renal profile and the associated outcome of patients with acute kidney injury undergoing dialysis in Renal Unit at a Tertiary Healthcare Facility in Western Kenya. Afri Health Sci.

[30] Zhang X, Gao C, Yin X (2025). Effect of modified Shenqi pill combined with conventional western medicine on Diabetic Nephropathy and its influence on Netrin-1 level and renal function. Afri Health Sci.

[31] Morais H, Cupessala VP, Pedro JM, Brito M, Gonçalves MAA (2025). Prevalence and factors associated with inadequate intake of fruits and vegetables in a population from Northern Angola. Afri Health Sci.

[32] Abd E-Kader SM, Refaey N, AlKhateeb AM, Al-Madaah M, Al-Fawaz SS, Neamatallah ZA (2025). Inflammatory cytokines and quality of life response to weight reduction in obese patients with bronchial asthma. Afri Health Sci.

[33] Han J (2025). Correlation between retention and masticatory ability of magnetic attachment overdenture in elderly patients. Afri Health Sci.

[34] Zhang Q, Zhou Z, Zhang L, Wang X, Zhao H, Wei C (2025). Phosphodiesterase 4 inhibitor combined with secukinumab relieves clinical symptoms in patients with psoriasis via regulating p38MAPK activation and the immune response inflammation. Afri Health Sci.

[35] Patel S, Nayak S, Chandrakar D, Wasnik PN, Jagzape TB, Mohapatra E (2025). Cytokine and immune cell interaction in immune-inflammatory response during crisis event in sickle cell disease. Afri Health Sci.

[36] Kou J, Li Q, Wang Y, Yue M, Yang S (2025). Association between frailty status and health literacy in the elderly. Afri Health Sci.

[37] Fubam RM, Olayemi O, Odukogbe A-TA, Tendongfor N (2025). Attitudes of secondary school adolescents towards premarital sexual activity and associated factors in Fako, Cameroon. Afri Health Sci.

[38] Makinde OI, Osegi N, Adesina AD, Atemie G, Ofuruma NN, Unachukwu CE (2025). Perceptions about reduced antenatal care contacts to a minimum of eight and its associated factors among pregnant women in Bayelsa State, Nigeria. Afri Health Sci.

[39] Zhang L, Wang H, Shen Y, Xie F, Xu M, Xiong B (2025). Relationship between sleep disorders and depression in perimenopausal women in the United States: a cross-sectional survey based on NHANES 2009-2014. Afri Health Sci.

[40] Rao K, Kumari S, Shetty SR, Babu SG, Castelino RL (2025). A clinical and biochemical study to estimate the salivary alpha-amylase level in post-menopausal women with psychosomatic disorders. Afri Health Sci.

[41] Andrew KF, Okpani A, A F (2025). Contraceptive prevalence and types used among female secondary students in public schools in Obio Akpor, Rivers State Nigeria. Afri Health Sci.

[42] Wang L, Cheng W, Zhao Y, Liu H (2025). Uterine artery chemoembolization compared with uterine artery embolization combined with prior to hysteroscopy and curettage in the treatment of cesarean scar pregnancy. Afri Health Sci.

[43] Shi C, Zong C, Yang J, Zhang H, Qi C, Li M (2025). Clinical analysis of Dexmedetomidine-Esketamine combined with intranasal administration before laparoscopic high ligation of hernia sac in infants and young children. Afri Health Sci.

[44] Wu X, Bian X, Zheng Q, Gu Y, Wang Y, Bao T (2025). Influencing factors of neural tube malformation: a systematic review and meta-analysis. Afri Health Sci.

[45] Sun T, Lin H (2025). Analysis of the use and advantages of depth of anaesthesia monitoring in children undergoing tonsillar adenoid surgery. Afri Health Sci.

[46] Filho JCOG, Rodrigues LFLR, Leite TB, Ribeiro da Silveira FD, Tapery CMC, Guimarães AS (2025). Prevalence of anxiety symptoms, depression and temporomandibular dysfunction in prisoners and workers from a Brazilian prison: an observational study. Afri Health Sci.

[47] Jaraczewski T, Diehl T, Jawara D, Tefera G, Zafar SN (2025). Surgical outcomes research in LMICs: a narrative review. Afri Health Sci.

[48] Tang G, Wang L, Jin G (2025). The value and safety of percutaneous vertebroplasty (PVP) for spinal compression fractures with the aid of G-arm fluoroscopy. Afri Health Sci.

